# Implementation of a Web Camera System in an Australian Neonatal Intensive Care Unit: Pre- and Postevaluation of the Parent and Staff Experience

**DOI:** 10.2196/47552

**Published:** 2023-11-22

**Authors:** Alexandra A Legge, Jennifer L Middleton, Shelley Reid, Adrienne Gordon

**Affiliations:** 1 Department of Newborn Care, Royal Prince Alfred Hospital, Camperdown, Sydney, Australia; 2 Susan Wakil School of Nursing and Midwifery, The University of Sydney, Sydney, Australia; 3 Sydney Institute of Women, Children and Families, Sydney Local Health District, Sydney, Australia; 4 Charles Perkins Centre, The University of Sydney, Sydney, Australia; 5 Faculty of Medicine and Health, The University of Sydney, Sydney, Australia

**Keywords:** web camera, telehealth, telemedicine, neonate, neonatology, NICU, virtual visitation, implementation, Australia, prematurity, illness, newborns, parent-infant separation, stress, parental, engagement, neonatal intensive care unit

## Abstract

**Background:**

Admission to a neonatal intensive care unit (NICU) for prematurity or illness is necessary for approximately 20% of newborns in Australia, resulting in parent-infant separation. Web cameras in the NICU provide a virtual link for parents to remain remotely connected to their infant during admission. Web camera use is increasing; however, there is limited evidence on the impact of web cameras on parents, infants, and neonatal staff.

**Objective:**

There were two objectives: (1) to determine the attitudes of parents and staff toward web cameras in the NICU and (2) to compare parental depression, anxiety, and stress levels using validated scales before and after web camera implementation in the NICU.

**Methods:**

A pre- and postevaluation survey was administered before and after implementation of the NICVIEW camera system in a tertiary NICU in Sydney, Australia. The NICVIEW camera system provides secure real-time viewing of infants and can be accessed from any device with an internet connection. Surveys were administered to parents of inpatients and staff, and included open- and closed-ended questions and Likert scales. Survey questions aimed to determine parent and staff attitudes and use of web cameras before and after implementation. In addition, pre- and postimplementation parental levels of depression, anxiety, and stress, as measured by the 21-item version of the Depression Anxiety Stress Scale (DASS-21) and Parental Stressor Scale: Neonatal Intensive Care Unit, were recorded.

**Results:**

In total, 94 parents and 109 staff members completed the pre- and postimplementation surveys. Post implementation, 43 of 44 (98%) parents supported web cameras, and 40 of 42 (95%) parents stated that they used web cameras. The most common reasons for support from parents included web cameras making parents feel more at ease, facilitating parent-infant bonding, increasing parental confidence in staff, and allowing others to see infants. There was no significant difference between the parental groups for the depression, anxiety, or stress scales measured by DASS-21. Staff support for web cameras increased significantly from 34 of 42 (81%) participants before to 64 of 67 (96%) participants after implementation (*P*=.01). Following implementation, there was a resolution in staff concerns about web cameras having an adverse impact on staff roles and privacy and security concerns.

**Conclusions:**

Web camera use in a tertiary Australian NICU was strongly supported by parents and staff and may reduce parental stress, facilitate parent-infant bonding, and encourage positive parent-staff engagement. Web cameras are a feasible method of providing continuity of care for families and should be considered as a standard of care in similarly resourced settings.

## Introduction

In Australia, approximately 1 in 5 babies require admission to a special care nursery or neonatal intensive care unit (NICU) [1], resulting in parent and infant separation. Advances in telecommunication and internet accessibility have allowed the implementation of web cameras into neonatal care units to facilitate virtual visitation. Web cameras are being increasingly used and provide parents with images, videos, or a live stream of their infant, accessible from any device with internet connectivity. Videophones were first used to connect parents remotely with their infants in 1983 [2], and the first internet web camera system Baby CareLink [3] was evaluated using a randomized trial in the late 1990s. Global uptake of the technology followed, with the first installation in Australia in 2009 [4]. Despite their widespread acceptance and use, there is limited evidence of the impact of web cameras on infants, families, and neonatal staff.

Bonding and attachment between parents and their infant may be interrupted when a neonatal admission occurs. An infant may need to stay in a neonatal unit geographically distanced from the family home for a period that may extend for months. Factors related to the NICU that may interfere with attachment and bonding include physical and logistical barriers to visiting, the physical environment of the NICU, parental stress, separation, and the intermittent sense of parenthood [5,6].

Web cameras provide one way for parents to remain connected to their infant when they are unable to be at the bedside and, thereby, mitigate factors that may disrupt bonding. This concept is supported by Dunham and Marin [7] who incorporated virtual visitation as a modifiable variable into a conceptual model describing the NICU maternal-infant bonding process. A recent systematic review found that web cameras may be helpful to reduce parent stress and anxiety, and enhance parental responsiveness and feelings of closeness with their infant, increasing emotional attachment [8]. This is supported by findings of reduced parental stress when given access to web cameras [9,10] and positive parental perceptions in the evaluation of a web camera system [9]. Web cameras are reported to be associated with improved breast milk–pumping experiences, increased motivation for mothers [11], and improved rates of breast milk feeding at the time of discharge from the NICU [12]. Other literature describes positive perceptions before the implementation of a web camera system [13] and the role of web cameras in assisting fathers in visiting their infants [14].

While attitudes toward web cameras are largely positive, staff working with web cameras have described increased workloads [15,16], disruptions to usual duties, and potential negative impacts on the quality of care [16]. Kubicka et al [9] did not find a significant difference in work-related burnout for staff who work with web cameras but described staff perceptions of increased nursing and parental stress, and no improvement in the quality of care provided to infants.

The Family Integrated Care (FICare) model of incorporating parents into caring for their infants in the NICU has demonstrated positive outcomes for infants [17] and reduced parental stress and anxiety [17,18]. Web cameras could be considered an extension of FICare for providing continuity for parents and virtual access to infants for other family and friends. The COVID-19 pandemic and its challenges, including limitations on visiting the NICU, have made it pertinent for NICUs to provide alternative ways for families and infants to remain connected.

This study aimed to determine parental and staff attitudes before and after the implementation of a web camera system in an Australian neonatal unit. A secondary aim was to determine parental depression, anxiety, and stress levels using validated scales before and after web camera implementation.

## Methods

### Design

The study is a pre- and postintervention evaluation survey administered to parents and staff before and after the installation of the NICVIEW camera system in one neonatal unit.

### Setting

The study was conducted in a tertiary neonatal unit located in a large metropolitan hospital in Sydney, Australia. The neonatal unit is an open-plan design of 10 intensive care beds and 24 beds for high- to low-dependency care. There are approximately 1000 admissions per year, of which approximately 50 are transferred from other hospitals.

### Intervention

The NICVIEW camera system was installed in June 2018. Above each bed space, a camera is located on an adjustable arm. The cameras provide secure real-time viewing of infants from any device with internet access. There is no audio component or storage of video footage. Parents are provided with verbal and written information, and are required to sign a consent form and accept the terms and conditions before use. Parents receive an email with unique log-in credentials to access the website. Log-in details can be shared with others at the parents’ discretion. Cameras are on 24 hours per day but turned off for episodes of care, procedures, and the relocation of patients to another area in the unit.

### Survey Instrument

Parent and staff surveys were developed by the authors for data collection. The parent survey included closed- and open-ended questions exploring demographics, visiting patterns (including barriers to visiting), camera use, and attitudes toward cameras. Parent data was collected in 5-year epoch categories. Gestational age at birth was converted from a continuous variable into categorical data consistent with stages of prematurity. As outlined below, validated scales were incorporated into the survey. The survey design was pragmatic, limiting the number of additional questions to minimize the survey burden. The staff survey included closed- and open-ended questions and Likert scales for exploring demographics, staff experience in using cameras, the cameras’ impact on their role, and staff support for or against cameras. The surveys were pilot-tested with a multidisciplinary group to ensure functionality and assess the timing for completion.

### Outcome Measures

The secondary aim was to assess parental depression, anxiety, and stress before and after web camera implementation; therefore, additional scales were used in the parent survey.

The 21-item version of the Depression Anxiety Stress Scale (DASS-21) [19] was administered to parents in the pre- and postimplementation surveys. The DASS-21 was used as a measure of the psychosocial impact of having an infant in the neonatal unit. It is a self-report instrument consisting of 3 scales of 7 items, measuring the emotional states of depression, anxiety, and stress. Respondents read each item and recorded their answers using a Likert scale ranging from 0 (did not apply to me at all) to 3 (applied to me very much or most of the time). Answers were summed to generate a total score for the depression, anxiety, and stress scales, and then stratified into mild, moderate, severe, and extremely severe categories. It has been validated for use in the general population, among pregnant women, and in the postpartum period [20-22].

Following analysis of the preimplementation results, the Parental Stressor Scale: Neonatal Intensive Care Unit (PSS:NICU) [23] was included in the postimplementation survey to gather comprehensive NICU-specific information about stress. The revised PSS:NICU was administered, consisting of 17 items in the “Looks and Behavior of the Infant” and 11 items in the “Parental Role” subscales. The “Looks and Behavior” subscale variables include parental perception of stress based on the appearance of the baby such as color, breathing, movement, and size; the use of equipment including intravenous lines; and behaviors of the baby, including crying and appearing to be sad or in pain. The “Parental Role” subscale variables include parental perception of stress to being separated from their baby; their role as a caregiver including feeding, touching, and providing care; and feelings of helplessness and of staff being closer to their baby. The camera system does not have audio; therefore, the “Sights and Sounds” subscale was omitted. All items were answered with a response scale of 1 (not stressful) to 5 (extremely stressful). PSS:NICU results were scored according to metric 2 (overall stress level), which considers all items to calculate the overall stress score. Items not experienced by the respondent were given a score of 1.

### Sample Size

The primary purpose of the study was to determine the attitudes of parents and staff before and after web camera implementation, with a secondary aim to determine any effect on DASS-21 and PSS:NICU scores. At the time of the study design, there were no data available to determine an expected estimate of the effect of DASS-21 or PSS:NICU scores in a population following web camera implementation. Therefore, a sample size was unable to be predetermined. We aimed to collect approximately 100 survey responses in both the parent and staff groups.

### Eligibility

All parents with an infant admitted to the neonatal unit during the preintervention (November 2017 and February 2018) and postintervention (July 2020 and May 2021) survey administration periods were eligible for inclusion. Parents of infants who had been discharged but admitted to the neonatal unit during the postimplementation period were eligible for inclusion in the postimplementation survey. There were no exclusion criteria based on gestation or multiple births or whether infants were inborn or transferred from other facilities.

All staff, including medical, nursing, allied health, and administrative staff, employed to work in the neonatal unit during the survey administration periods were eligible for inclusion.

Parent and staff surveys were only available for completion in the English language.

### Recruitment and Data Collection

The preimplementation parent survey was distributed to parents of infants admitted to the NICU between November 2017 and February 2018. The postimplementation survey was distributed to parents of infants admitted between July 2020 and May 2021 following camera installation. Surveys were distributed using flyers with QR codes or website links, and paper copies were available for parents to complete at a convenient time. During their infant’s admission, parents of inpatients were approached once by designated staff not providing clinical care to the infant to request survey completion. As the postimplementation surveys were distributed during the COVID-19 lockdown, which affected the number of visitors to the NICU, the QR code and website links to the postimplementation survey were published in a newsletter distributed to parents of infants who were recently discharged from the neonatal unit but admitted during the postimplementation period.

The staff survey was distributed for completion between August and September 2017 before camera installation and between July and November 2020 after installation. Surveys were distributed using flyers with QR codes and a website link, emails with a website link, and paper copies. Staff were approached by authors and designated staff to request survey completion in person and by email during the study period.

### Data Analysis

Study data were collected and managed using REDCap, an electronic data capture tool hosted at Sydney Local Health District [24,25]. REDCap is a secure web-based software platform designed to support data capture for research studies. Results were exported from REDCap into Excel version 16.49 (Microsoft Corporation) for provisional analysis, and descriptive statistics were generated using SPSS Statistics 27 (IBM Corp). Parent and staff demographic and web camera characteristics were described using proportions and compared using chi-square and Fisher exact tests. DASS-21 ordinal scores were assessed for distribution and investigated using the Mann-Whitney *U* test. *P* values less than .05 were considered statistically significant. We planned to perform a multivariate analysis if any univariate associations were significant; however, no significant associations were noted in the univariate analysis.

### Ethical Considerations

Consent was implied by survey completion following an introductory consent statement. Surveys could be discontinued at any stage. All surveys were anonymous unless parents or staff chose to provide details. No respondents received compensation for participation in the survey.

The study was approved by the Ethics Review Committee (Royal Prince Alfred Hospital Zone) of the Sydney Local Health District (protocol X16-0298 & HREC/16/RPAH/381).

## Results

### Overview

A total of 125 parents and 110 staff members commenced the surveys. Data analyses were possible for ~94 parents and 109 staff members with complete responses. The exact response rate for the survey is uncalculable as the survey was anonymous, and twins and multiple parents of the same infant are unable to be accounted for. In addition, families who declined or were not offered a web camera in the postimplementation period were not documented. A total of 301 babies were admitted in the preimplementation and 941 in the postimplementation period.

### Parents

#### Demographics

Parent demographic characteristics for parents who completed the survey are presented in Table 1. There was no significant difference between groups. We also compared parent demographic characteristics for parents who did not complete the survey and noted no differences (Multimedia Appendix 1).

**Table 1.** Characteristics of parents in the pre- and postimplementation web camera groups.

Characteristic		Preimplementation (n=43), n (%)	Postimplementation (n=51), n (%)	*P* value
**Gender**				.63
	Female	34 (79)	38 (75)	
	Male	9 (21)	13 (26)	
**Age (years**)				.24
	20-24	2 (5)	2 (4)	
	25-29	4 (9)	3 (6)	
	30-34	13 (30)	25 (49)	
	35-39	12 (28)	14 (28)	
	40-44	9 (21)	7 (14)	
	≥45	3 (7)	0 (0)	
**Marital status**				.43
	Married or de facto	37 (86)	46 (90)	
	Never married	3 (7)	3 (6)	
	Separated	0 (0)	2 (4)	
	Widowed	0 (0)	0 (0)	
	Did not answer	3 (7)	0 (0)	
**Country of birth**				.22
	Born in Australia	29 (67)	28 (55)	
	Born overseas	14 (33)	23 (45)	
**Language spoken**				.35
	English only	34 (79)	36 (71)	
	Additional language	9 (21)	15 (29)	
**Highest level of education**				.29
	Postgraduate degree	15 (35)	16 (31)	
	Bachelor’s degree	15 (35)	21 (41)	
	Certificate, diploma, or advanced diploma	6 (14)	6 (12)	
	Graduate certificate	1 (2)	4 (8)	
	High school	6 (14)	2 (4)	
	Did not finish high school	0 (0)	2 (4)	
**Employment status**				.11
	Full-time	21 (49)	29 (57)	
	Part-time	11 (26)	3 (6)	
	Do not have a job	2 (5)	5 (10)	
	On paid leave	4 (9)	13 (26)	
	Other	4 (9)	1 (2)	
	Did not answer	1 (2)	0 (0)	
**Place of birth**				.10
	Inborn	35 (81)	48 (94)	
	Ex utero transfer	8 (19)	3 (6)	
**Gestation (weeks**)				.21
	24-25	2 (5)	2 (4)	
	26-27	4 (9)	2 (4)	
	28-31	22 (51)	17 (33)	
	32-36	12 (28)	22 (43)	
	37-42	3 (7)	8 (16)	

#### Parental Visiting and Barriers

Most parents reported that they visited their infant daily. A higher proportion of parents experienced barriers to visiting after web camera implementation (13/43, 30%) compared to preimplementation (7/42, 17%). There was a reduction from pre- to postimplementation in the proportion of parents who stated they would feel better if they could visit for longer. Detailed results for parental visiting and barriers are outlined in Table 2.

**Table 2.** Parental visiting and barriers for the pre- and postimplementation web camera groups.

		Preimplementation, n (%)	Postimplementation, n (%)	*P* value
**Visiting frequency**				>.99
	Daily	34 (92)	38 (93)	
	3-5 d/wk	3 (8)	3 (7)	
	Total	37	41	
**Time (hours/visit)**				.99
	>8	8 (21)	7 (17)	
	5-8	17 (44)	20 (48)	
	3-4	11 (28)	13 (31)	
	1-2	3 (8)	2 (5)	
	<1	0 (0)	0 (0)	
	Total	39	42	
**Would you feel better if you could visit for longer?**				.01
	Yes	33 (81)	23 (55)	
	No	8 (20)	19 (45)	
	Total	41	42	
**Barriers to visiting experienced**				.14
	Yes	7 (17)	13 (30)	
	No	35 (83)	30 (70)	
	Total	42	43	
**Barriers identified**				N/A^a^
	Personal health	1 (14)	3 (23)	
	Lack of transport	3 (43)	6 (46)	
	Financial	1 (14)	1 (8)	
	Caring for other children	3 (43)	7 (54)	
	Distance required to travel	0 (0)	5 (39)	
	Work commitments	1 (14)	0 (0)	
	COVID-19 reasons	0 (0)	5 (39)	
	Other	0 (0)	2 (15)	
	Total	7	13	

a N/A: not applicable.

#### Parental Web Camera Use and Attitudes

Web cameras were reported to be used by the majority of parents (40/42, 95%), and most parents (30/42, 71%) stated that their family used the web camera. The most common viewing times were in the evening and overnight. Almost all parents were supportive of web cameras before (37/40, 92%) and after (43/44, 98%) implementation. The most common reason for support in both groups was that the web camera helped parents feel at ease when they were unable to visit their infant. After implementation, one parent was not supportive of web cameras, identifying that they made them feel anxious or stressed. Concerns regarding privacy and security were not cited as reasons against support after implementation. Detailed results for parental views are outlined in Table 3, and free-text comments from parents in the postimplementation survey are included in Multimedia Appendix 2.

**Table 3.** Parental views on web cameras in the pre- and postimplementation groups.

		Preimplementation, n (%)	Postimplementation, n (%)	*P* value
**Support web camera use**				.34
	Yes	37 (93)	43 (98)	
	No	3 (7)	1 (2)	
	Total	40	44	
**Reasons for web camera support**				N/A^a^
	Parent-infant bonding	16 (43)	21 (49)	
	Help parents feel at ease if unable to visit	36 (97)	37 (86)	
	Allow others to see infant	27 (73)	29 (67)	
	Increase confidence in staff	20 (54)	23 (54)	
	Positive staff engagement	18 (49)	17 (40)	
	Breastfeeding/expressing	0 (0)	3 (7)	
	Transition to home	0 (0)	1 (2)	
	Other	3 (8)	1 (2)	
	Total	37	43	
**Reasons against web camera support**				N/A
	Compromise parental care	1 (33)	0 (0)	
	Privacy concerns	3 (100)	0 (0)	
	Security concerns	1 (33)	0 (0)	
	Distraction from care	1 (33)	0 (0)	
	Increase anxiety or stress	3 (100)	1 (100)	
	Witness adverse event or infant distress	2 (67)	1 (100)	
	Total	3	1	

a N/A: not applicable.

#### Parental Depression, Anxiety, and Stress

There were no significant differences between the groups for the DASS-21 depression, anxiety, or stress scales (Table 4). However, there was a nonsignificant trend toward lower stress scales as measured by DASS-21 in the postimplementation group. The PSS:NICU scores were collected for the postimplementation group (Table 4) and provided NICU-specific information regarding stress for parents.

**Table 4.** Parent Depression Anxiety Stress Scale (DASS-21) scores for the pre- and postimplementation web camera groups and Parental Stressor Scale: Neonatal Intensive Care Unit (PSS:NICU) mean scores for the parent postimplementation group.

				Preimplementation	Postimplementation	*P* value
**DASS 21, n (%)**						
	**Depression**					.83
		Normal		34 (79)	40 (78)	
		Mild		4 (9)	7 (14)	
		Moderate		5 (12)	3 (6)	
		Severe		0 (0)	1 (2)	
		Extremely severe		0 (0)	0 (0)	
		Total		43	51	
	**Normal vs any depression**					.94
		Normal		34 (79)	40 (78)	
		Any depression		9 (21)	11 (22)	
		Total		43	51	
	**Anxiety**					.42
		Normal		29 (67)	35 (69)	
		Mild		8 (19)	8 (16)	
		Moderate		1 (2)	5 (10)	
		Severe		0 (0)	1 (2)	
		Extremely severe		5 (12)	2 (4)	
		Total		43	51	
	**Normal vs any anxiety**					.90
		Normal		29 (67)	35 (69)	
		Any anxiety		14 (33)	16 (31)	
		Total		43	51	
	**Stress**					.48
		Normal		28 (65)	37 (73)	
		Mild		9 (21)	8 (16)	
		Moderate		3 (7)	4 (8)	
		Severe		2 (5)	2 (4)	
		Extremely severe		1 (2)	0 (0)	
		Total		43	51	
	**Normal vs any stress**					.44
		Normal		28 (65)	37 (73)	
		Any stress		15 (35)	14 (28)	
		Total		43	51	
**PSS:NICU, mean (SD)**						N/A^a^
	Looks and behavior (n=47)			N/A	2.65 (1.50)	
	Parental role (n=49)			N/A	2.74 (1.35)	

a N/A: not applicable.

### Staff

Staff characteristics are shown in Table 5. Staff support for web cameras significantly increased from 34 of 42 (81%) participants supporting before implementation to 64 of 67 (96%) participants after implementation (*P*=.01). There was a reduction in staff concerns regarding the web cameras having an adverse impact on their role in the postimplementation survey. The main areas for staff concern included the web cameras taking time away from patient care, technical or equipment issues relating to web cameras, phone calls from parents, and parental anxiety. A total of 15 staff members identified that they would prefer restrictions on the time the web cameras can be turned on, with the majority stating that cameras should be turned off during procedures or caring routines. The majority of staff members (54/67, 81%) found current web camera resources adequate. Staff suggested additional support resources including a guideline or mobile app. Staff views on the web cameras and their impact on the staff’s role are outlined in Table 6.

**Table 5.** Characteristics of staff in the pre- and postimplementation web camera groups.

		Preimplementation, n (%)	Postimplementation, n (%)	*P* value
**Role**				.60
	Administration	1 (2)	1 (2)	
	Nursing	37 (88)	63 (93)	
	Medical	4 (10)	3 (4)	
	Allied health	0 (0)	1 (2)	
	Total	42	68	
**Time worked in a tertiary neonatal setting (years)**				.24
	<1	2 (5)	5 (8)	
	1-5	10 (24)	25 (37)	
	6-10	6 (14)	12 (18)	
	>10	24 (57)	25 (37)	
	Total	42	67	
**Time worked at study site (years)**				.24
	<1	3 (7)	9 (13)	
	1-5	11 (26)	25 (37)	
	6-10	5 (12)	10 (15)	
	>10	23 (55)	24 (35)	
	Total	42	68	
**Age (years)**				.33
	<25	1 (2)	3 (5)	
	25-35	16 (38)	37 (55)	
	36-45	12 (29)	12 (18)	
	46-55	5 (12)	8 (12)	
	>55	8 (19)	7 (10)	
	Total	42	67	
**Born in Australia**				.20
	Yes	24 (59)	46 (71)	
	No	17 (42)	19 (29)	
	Total	41	65	
**Area worked**				.98
	Intensive care	37 (88)	56 (82)	
	High dependency	37 (88)	59 (87)	
	Special care nursery	36 (86)	51 (75)	
	Outpatients	3 (7)	5 (7)	
	Total	42	68	
**Previous web camera experience**				.67
	Yes	3 (7)	3 (4)	
	No	39 (93)	65 (96)	
	Total	42	68	

**Table 6.** Staff views on web cameras in the pre- and postimplementation web camera groups.

		Preimplementation, n (%)	Postimplementation, n (%)	*P* value
**Support web cameras**				.01
	Yes	34 (81)	64 (96)	
	No	8 (19)	3 (5)	
	Total	42	67	
**Reasons for support**				N/A^a^
	Parent-infant bonding	17 (50)	47 (73)	
	Reduce parental anxiety	31 (91)	59 (92)	
	Allow others to see infant	24 (71)	58 (91)	
	Increase confidence in staff and the unit	16 (47)	26 (41)	
	Positive parental-staff engagement	20 (59)	31 (48)	
	Other	4 (12)	4 (6)	
	Total	34	64	
**Reasons against support**				N/A
	Physically impede access	3 (38)	1 (33)	
	Compromise care	1 (13)	1 (33)	
	Privacy concerns	8 (100)	0 (0)	
	Security concerns	5 (63)	0 (0)	
	Distraction from care	5 (63)	3 (100)	
	Staff anxiety/stress	4 (50)	0 (0)	
	Parents witness adverse event or infant distress	5 (63)	2 (67)	
	Reduced parental visiting	4 (50)	0 (0)	
	Other	6 (75)	2 (67)	
	Total	8	3	
**Adverse impact on staff role**				N/A
	Strongly agree	2 (5)	1 (2)	
	Agree	7 (17)	7 (10)	
	Unsure	10 (24)	13 (19)	
	Disagree	16 (39)	34 (51)	
	Strongly disagree	6 (15)	12 (18)	
	Total	41	67	

a N/A: not applicable.

## Discussion

### Principal Results

Our pre-post implementation study found that web camera use in an Australian tertiary neonatal unit was strongly supported by both parents and staff, and that web cameras were well used following installation. Web cameras may assist with reducing parental stress and facilitating parent-infant bonding while not increasing parental reported depression or anxiety. Initial staff concerns about web cameras were largely alleviated following their implementation. Parent confidence in staff and positive staff engagement were common reasons cited for web camera support from parents in both the pre- and postimplementation periods.

### Comparison With Prior Work

This study simultaneously examines parent and staff perceptions, and the impact of web cameras on parent depression, anxiety, and stress in neonatal care. Our findings support the existing conclusion that web cameras are an acceptable and feasible intervention to facilitate virtual visitation and support families during a neonatal admission [26-28].

DASS-21 scores demonstrated that the implementation of web cameras did not increase parents’ levels of depression, anxiety, or stress in this study. There was, however, a nonsignificant trend in reducing any stress on DASS-21 post implementation (15/43, 35% preimplementation to 14/51, 28% postimplementation). We investigated this trend further by comparing the mean PSS:NICU subscale scores (collected in the postimplementation survey) with scores from a published comparison population group enrolled in the FICare intervention trial. This trial, completed in Canada, Australia, and New Zealand, aimed to identify how FICare affects maternal stress and anxiety, and has published baseline data from similar neonatal units. Our study’s PSS:NICU scores for “Looks and Behavior” (mean 2.65, SD 1.50 vs mean 2.78 [18], mean difference −0.13, 95% CI −0.23 to −0.02; *P*=.02) and “Parental Role” (mean 2.74, SD 1.35 vs mean 2.98 [18], mean difference −0.23, 95% CI −0.35 to −0.12; *P*<.001) were significantly lower when compared to the group in Cheng et al [18]. This comparison, although not within our setting, supports the concept that a web camera service within a NICU may reduce parental stress. This result is further supported by recent findings by Kubicka et al [9] who found a reduction in PSS:NICU scores comparing an “on web camera” to an “off web camera” group.

Reasons for web camera support identified by parents included that web cameras help with bonding, make parents feel more at ease, and allow others to see the infant. Kubicka et al [9] discussed similar findings, with 86% of parents reporting that watching their baby on the web camera made them feel better. These findings are further supported by Kerr et al [28] who highlighted the positive parental perceptions of web cameras, including enhanced feelings of closeness, emotional well-being, and the involvement of family and friends. A small number (n=3) of parents in this study identified that web cameras helped with breastfeeding and expressing. A sustained intention to breastfeed or provide breast milk to the baby and an improved expressing experience has been described in recent literature [11,12], which provides a positive direction for future research.

Our findings indicate that, following implementation, most staff support the use of web cameras. This finding is in contrast to those by Kubicka et al [9] who reported that 86% of the nursing staff did not believe that web cameras improved infants’ quality of care. Our findings suggest that initial staff concerns regarding web cameras are balanced by experience and the identified benefits for families, a sentiment previously discussed by Joshi et al [16]. Furthermore, staff should be reassured that many parents identified that web cameras increased their confidence in and had positive effects on their engagement with staff. This was also the case for the Baby CareLink intervention group, which reported higher overall quality of care [3], and for Kubicka et al [9], who reported that 83% of parents were reassured about their infant’s nursing care. The hesitations regarding the privacy and security of web cameras were not sustained following implementation. However, a small number of staff reported that web cameras may have an adverse impact on their role, highlighting that support for and provision of adequate resources for staff are imperative for the ongoing success of a web camera service.

### Strengths and Limitations

Strengths of our study included the pre-post implementation approach, the inclusion of both parents and staff, and the use of validated scales to assess depression, anxiety, and stress. Our NICU is located in a large Australian city and services a culturally diverse population, increasing the generalizability of results to other tertiary neonatal units. The separation in periods between the pre- and postsurvey administration allowed for web cameras to be well integrated within the culture of the neonatal unit. A study limitation was the absence of preimplementation PSS:NICU results. This scale was added to the postimplementation survey, as the analysis of the preimplementation DASS-21 scores suggested a reduction in stress, and the PSS:NICU collects NICU-specific stress information. We addressed this limitation by using a large comparison cohort that was generalizable to our study population [18] to compare our scores. Additional limitations include the absence of a prespecified sample size specifically for the DASS-21 outcomes for parents and the unlikely but possible potential for duplicate survey responses. The timing of survey completion by parents during their infant’s admission was not collected and may be a confounder. There is potential reporter bias, as those parents completing the survey may be more likely to use and be supportive of the cameras; however, our respondents appeared to be representative of our unit’s parent population. The postimplementation survey was administered during the COVID-19 pandemic when restrictions limited visits to one parent at a time. We hypothesize that support for web cameras will inevitably increase during a period where visiting is limited, and this may potentially reduce the generalizability of results.

### Conclusions

This study contributes to the growing body of evidence on the impact of web cameras in NICUs. Web cameras were strongly supported by parents and staff, and may reduce parental stress, facilitate parent-infant bonding, and encourage positive parent-staff engagement.

Web cameras are a feasible and acceptable method of providing support and continuity of care for families during neonatal unit admission and should be considered as a standard of care in similarly resourced settings.

## Supplementary material

10.2196/47552 Multimedia Appendix 1 Characteristics of parents for complete and incomplete surveys for the pre- and postimplementation of web camera groups.

10.2196/47552 Multimedia Appendix 2 Free-text parent comments from the postimplementation survey.

## Abbreviations

DASS-21: 21-item version of the Depression Anxiety and Stress Scales
FICare: Family Integrated Care
NICU: neonatal intensive care unit
PSS:NICU: Parental Stressor Scale: Neonatal Intensive Care Unit
