# Supplementary material for: Implementation of a Web Camera System in an Australian Neonatal Intensive Care Unit: Pre- and Postevaluation of the Parent and Staff Experience
Source: JMIR Pediatr Parent. 2023 Nov 22;6:e47552. doi: 10.2196/47552 (PMC10690101; doi:10.2196/47552)
Supplement: Multimedia Appendix 1 [file pediatrics-v6-e47552-s001.docx]

Appendix 1. Characteristics of parents for complete and incomplete surveys for pre- and postimplementation of webcamera groups.

|  | Character-istic | Pre  Implement-ation (Complete) | Pre implement-ation (Incomplete) | *P* | Post  Implement-ation (Complete) | Post implement-ation (Incomplete) | *P* |
| --- | --- | --- | --- | --- | --- | --- | --- |
|  |  | n (%) | n (%) |  | n (%) | n (%) |  |
|  |  |  |  |  |  |  |  |
| **Gender** |  |  |  |  |  |  |  |
|  | Female | 34 (79.1) | 21 (95.5) | .14 | 38 (74.5) | 5 (55.6) | .26 |
|  | Male | 9 (20.9) | 1 (4.5) |  | 13 (25.5) | 4 (44.4) |  |
|  | *Total* | 43 | 22 |  | 51 | 9 |  |
| **Age (years)** |  |  |  |  |  |  |  |
|  | 20-24 | 2 (4.7) |  | .17 | 2 (3.9) |  | .16 |
|  | 25-29 | 4 (9.3) |  |  | 3 (5.9) | 1 (11.1) |  |
|  | 30-34 | 13 (30.2) | 11 (50.0) |  | 25 (49.0) | 2 (22.2) |  |
|  | 35-39 | 12 (27.9) | 5 (22.7) |  | 14 (27.5) | 3 (33.3) |  |
|  | 40-44 | 9 (20.9) | 2 (9.1) |  | 7 (13.7) | 2 (22.2) |  |
|  | 45+ | 3 (7.0) | 4 (18.2) |  |  | 1 (11.1) |  |
|  | *Total* | 43 | 22 |  | 51 | 9 |  |
| **Marital status** |  |  |  |  |  |  |  |
|  | Married or de facto | 37 (86.0) | 16 (72.7) | .05 | 46 (90.2) | 9 (100) | .61 |
|  | Never married | 3 (7.0) | 2 (9.1) |  | 3 (5.9) |  |  |
|  | Separated |  | 3 (13.6) |  | 2 (3.9) |  |  |
|  | Widowed |  | 1 (4.5) |  |  |  |  |
|  | Did not answer | 3 (7.0) |  |  |  |  |  |
|  | *Total* | 43 | 22 |  | 51 | 9 |  |
| **Country of birth** |  |  |  |  |  |  |  |
|  | Born in Australia | 29 (67.4) | 10 (45.5) | .87 | 28 (54.9) | 3 (33.3) | .29 |
|  | Born overseas | 14 (32.6) | 12 (54.5) |  | 23 (45.1) | 6 (66.7) |  |
|  | *Total* | 43 | 22 |  | 51 | 9 |  |
| **Language spoken** |  |  |  |  |  |  |  |
|  | English only | 34 (79.1) | 14 (63.6) | .18 | 36 (70.6) | 5 (55.6) | .45 |
|  | Additional language | 9 (20.9) | 8 (36.4) |  | 15 (29.4) | 4 (44.4) |  |
|  | *Total* | 43 | 22 |  | 51 | 9 |  |
| **Highest level of education** |  |  |  |  |  |  |  |
|  | Postgraduate degree | 15 (34.9) | 6 (27.3) | .56 | 16 (31.4) | 4 (44.4) | .99 |
|  | Bachelor’s degree | 15 (34.9) | 8 (36.4) |  | 21 (41.2) | 4 (44.4) |  |
|  | Certificate, diploma or advanced diploma | 6 (14.0) | 6 (27.3) |  | 6 (11.8) |  |  |
|  | Graduate certificate | 1 (2.3) | 2 (9.1) |  | 4 (7.8) |  |  |
|  | High school | 6 (14.0) |  |  | 2 (3.9) | 1 (11.1) |  |
|  | Did not finish high school |  |  |  | 2 (3.9) |  |  |
|  | *Total* | 43 | 22 |  | 51 | 9 |  |
| **Employment status** |  |  |  |  |  |  |  |
|  | Full-time | 21 (48.8) | 8 (36.4) | .19 | 29 (56.9) | 9 (100) | .19 |
|  | Part-time | 11 (25.6) | 4 (18.2) |  | 3 (5.9) |  |  |
|  | Do not have a job | 2 (4.7) | 3 (13.6) |  | 5 (9.8) |  |  |
|  | On paid leave | 4 (9.3) | 4 (18.2) |  | 13 (25.5) |  |  |
|  | Other | 4 (9.3) | 3 (13.6) |  | 1 (2.0) |  |  |
|  | Did not answer | 1 (2.3) |  |  |  |  |  |
|  | *Total* | 43 | 22 |  | 51 | 9 |  |
| **Place of birth** |  |  |  |  |  |  |  |
|  | Inborn | 35 (81.4) | 21 (95.5) | .15 | 48 (94.1) | 9 (100) | >.99 |
|  | Ex utero transfer | 8 (18.6) | 1 (4.5) |  | 3 (5.9) |  |  |
|  | *Total* | 43 | 22 |  | 51 | 9 |  |
| **Gestation (weeks)** |  |  |  |  |  |  |  |
|  | 24-25 | 2 (4.7) | 2 (9.1) | .63 | 2 (3.9) |  | .83 |
|  | 26-27 | 4 (9.3) | 2 (9.1) |  | 2 (3.9) |  |  |
|  | 28-31 | 22 (51.2) | 7 (31.8) |  | 17 (33.3) | 2 (22.2) |  |
|  | 32-36 | 12 (27.9) | 9 (40.9) |  | 22 (43.1) | 5 (55.6) |  |
|  | 37-42 | 3 (7.0) | 2 (9.1) |  | 8 (15.7) | 2 (22.2) |  |
|  | *Total* | 43 | 22 |  | 51 | 9 |  |
