# Supplementary material for: Implementation of a Web Camera System in an Australian Neonatal Intensive Care Unit: Pre- and Postevaluation of the Parent and Staff Experience
Source: JMIR Pediatr Parent. 2023 Nov 22;6:e47552. doi: 10.2196/47552 (PMC10690101; doi:10.2196/47552)
Supplement: Multimedia Appendix 2 [file pediatrics-v6-e47552-s002.docx]

Appendix 2. Free-text parent comments from post implementation survey.

| I think it helps reduce my wife's anxiety and guilt when she's away from our baby.  Making sure the camera is on after a care or constant check if it is on during their shift does make a parent less anxious knowing their little one is alright by visualising them on the camera.  I don't think the camera should be switched off when nurses are caring for the baby. Even looking & seeing an empty cot would be ok as you can infer what is happening.  I really love the use of cameras and as someone who was a bit anxious about my child in NICU, it is reassuring that you are able to view the camera. The only real improvement would be in image quality - it's just quite grainy.  It really helps me relax in the evenings when I'm away from baby and my family love it as they can see baby whenever they want.    It was a little bittersweet. It was great to be able to see my baby overnight when I couldn't be there, but at the same time it was heartbreaking when I would log on and see him crying. It made me feel very sad that I could not be there for him and could not hold or comfort him.  … the cameras have provided invaluable benefits in allowing us to stay connected to our babies if we can't be there with them.  It was great! I live 5hrs away from the hospital my baby was at. My husband stayed at home to care for our older daughter so it was great for him to be able to look at her when he couldn't be with us.    I use the camera when I sit down to pump and 1st thing in the morning. Cameras give you the opportunity to see the baby when unable to visit.  Our families use the cameras a lot and it’s been nice knowing they can log on to see the babies.  Due to the humidity the camera is always blurry. It would be great if we can skew the camera remotely via Web as not to bother nurses.  ... I always feared the worst when the cameras were switched off, it really adds to the stress and anxiety.  Amazing! I feel so lucky to be able to see my baby when I'm not near by.  The camera was amazing to have and made a tough time much easier. |
| --- |
